# Subacromial Impingement Syndrome of the Shoulder: A Musculoskeletal Disorder or a Medical Myth?

**DOI:** 10.5704/MOJ.1911.001

**Published:** 2019-11

**Authors:** KS Dhillon

**Affiliations:** Department of Orthopaedics, KPJ Selangor Specialist Hospital, Shah Alam, Malaysia

**Keywords:** subacromial impingement syndrome, acromioplasty, subacromial decompression, medical myth

## Abstract

Subacromial impingement syndrome (SAIS) is a commonly diagnosed disorder of the shoulder. Though this disorder has been known for a long time, it remains a poorly understood entity. Over the years several hypotheses have been put forward to describe the pathogenesis of SAIS but no clear explanation has been found. Two mechanisms, the extrinsic and intrinsic mechanism, have been described for the impingement syndrome. The intrinsic mechanism theories which deny the existence of impingement are gaining popularity in recent years.

The various shoulder tests used to diagnose SAIS have low specificity with an average of about 50%. Meta-analysis shows that neither the Neer sign nor the Hawkins sign has diagnostic utility for impingement syndrome.

Several randomised controlled trials have shown that the outcome of treatment of SAIS by surgery is no better than conservative treatment. Physiotherapy alone can provide good outcome which is comparable to that achieved with surgery without the costs and complications associated with surgery.

Since decompression with surgery does not provide any additional benefits as compared to conservative treatment for patients with SAIS, the impingement theory has become antiquated and surgical treatment should have no role in the treatment of such patients. There are calls by some practitioners to abandon the term impingement syndrome and rename it as anterolateral shoulder pain syndrome. It appears that SAIS is a medical myth. There are others who called SAIS as a clinical illusion.

## Introduction

The subacromial space lies between the coracoacromial arch above and the humeral head and greater tuberosity of the humerus below. It contains the rotator cuff tendons, the long head of biceps tendon, the shoulder joint capsule, the glenohumeral ligament, coraco-humeral ligament and the subacromial bursa.

Subacromial pathology has attracted the attention of orthopaedic surgeons for a long time. In 1934, Codman^1^ described rotator cuff pathology and he was of the opinion that humeral head and acromion impingement during shoulder abduction was the cause of rotator cuff lesions and he suggested that lateral acromioplasty would resolve the patient's symptoms.

In 1972, Neer coined the term impingement syndrome and he was of the opinion that impingement occurred anterolaterally at the anterior acromion and the coracoacromial ligament^2^. He proposed anterior acromioplasty as a mode of treatment for impingement syndrome.

Subacromial impingement syndrome (SAIS) of the shoulder is probably the most common disorder of the shoulder and accounts for about 48% of all shoulder complaints^3^.

There has been a dramatic increase in the incidence of acromioplasty over the years. Vitale *et al*^4^ examined the American Board of Orthopaedic Surgery database from 1999 to 2008 and found a 142.3% increase in the number of arthroscopic acromioplasties performed during the period. These figures raise the question of the role of such surgery in patients with SAIS and whether such surgery is justified in patients with SAIS.

### What is Subacromial Impingement Syndrome?

In 1972, Dr Charles Neer^2^ introduced the idea that rotator cuff problems were the result of contact or “impingement” of the rotator cuff tendons to the acromion, the coracoacromial ligament or the undersurface of the acromioclavicular joint. Subacromial impingement syndrome (SAIS) now includes a spectrum of subacromial space pathologies which include rotator cuff tears, calcific tendinitis, biceps tendinopathy, rotator cuff tendinosis and subacromial bursitis^5^.

The aetiology of SAIS remains a mystery. Different hypotheses have been put forward to describe the pathogenesis of SAIS but no clear explanation has yet been found.

Two mechanisms, the extrinsic and intrinsic mechanisms, have been proposed for the genesis of the impingement syndrome. In the intrinsic mechanism, it is believed that damage to the rotator cuff tendons leads to impingement and in the extrinsic mechanism the impingement is believed to cause damage to the tendons^6, 7^. The intrinsic mechanism theories are gaining popularity in recent years^8, 9^.

As is the case with the tendo Achilles, the supraspinatus tendon too has poor vascularity near its insertion on the greater tuberosity. There is an avascular zone called the critical zone and it is here that the degenerative tears of the supraspinatus tendon originate^7^. The damage to the tendon fibres increases in size as we age, and the damage is more common in patients who are diabetics^10^. Histological examination of the tendon often shows a “failed healing response”^8, 11^.

Proponents of the extrinsic mechanism tried to correlate shoulder pain to shoulder “impingement”. Hooked (type III) acromions were believed to cause “impingement” as a result of the reduced distance in the subacromial space. To date, however, it is not known whether the shape of the acromion is age-related or congenital^12^.

A protracted scapula and weakness of the scapular muscles, particularly the serratus anterior and trapezius have also been implicated in shoulder impingement. Abnormality of the glenohumeral joint and weakness of the rotator cuff muscles can lead to superior migration of the humeral head which can cause impingement as well.

Several other extrinsic factors including, heavy physical loading, vibration, injury, smoking, infection, genetic factors, and fluoroquinolones^13^ have also been implicated in the genesis of rotator cuff disease and shoulder pain. According to Neer, trauma may enlarge a rotator cuff tear but is rarely the principal factor^14^.

### Diagnosis of Subacromial Syndrome

The history of patients with SAIS is usually consistent. They complain of shoulder pain which usually develops insidiously over a period of weeks to months. The pain is usually localised anterior and lateral to the acromion and frequently radiates to the lateral aspect of the mid-arm. The pain is more common at night and is exacerbated by lying on the involved side and sleeping with the arm overhead. Overhead activities produce pain in the shoulder. Sometimes weakness and stiffness may be present due to pain.

There are several clinical tests used to diagnose SAIS such as the Neer test, Hawkins-Kennedy test, Impingement test, Drop arm test, and Jobe's test. The specificity for these tests is poor. The average specificity for the Neer’s test is about 36±22% and for the Hawkins sign, the specificity is about 41±19%^15^. Hegedus *et al*^16^ in their meta-analysis concluded that neither the Neer nor the Hawkins sign had diagnostic utility for impingement syndrome.

Imaging studies are of not much value in elucidating the cause of shoulder pain and in the diagnosis of SAIS. An MRI will show rotator cuff pathology and bursitis but it will not pinpoint the cause of shoulder pain.

### Treatment of Subacromial Impingement Syndrome

**A. Non-Operative Treatment**

A review of the literature by Bigliani and Levine^17^ in 1997 showed that most patients with shoulder impingement syndrome eventually recover with non-operative intervention^18-21^. The most common non-operative treatment modalities used include modification of activity, the use of non-steroidal anti-inflammatory drugs, subacromial steroid injections and physical therapy programs^17^.

Morrison *et al*^22^ retrospectively reviewed the outcome of conservative treatment of 616 patients (636 shoulders) who had subacromial impingement syndrome. All the patients were treated with NSAIDs and isometric and isotonic muscle strengthening exercises. The average follow-up period was 27 months. Sixty seven percent of the patients had a satisfactory outcome, 28% had an unsatisfactory outcome and were treated with surgical decompression and 5% of the patients had an unsatisfactory outcome but they declined surgical intervention.

The outcome was better in patients who were 20 years old or less and those who were between 41 years to 60 years old as compared to those who were 21 years to 40 years of age. The outcome was worse in patients who were more than 60 years of age. The outcome was better in patients with type-I acromion as compared to patients with type-II or type-Ill acromion.

Hanratty *et al*^23^ carried out a systematic review and meta-analysis to assess the effectiveness of exercises in SAIS. They found that there was strong evidence that exercise decreases pain and improves function on short-term follow-up. They also found moderate evidence that exercise results in short-term improvement in mental well-being and long-term improvement in function.

The exercise programs include scapular stabilisation exercises, rotator cuff resistance exercises, range of motion as well as stretching exercises. Of these exercise programs which is most effective is not known^23, 24^. There is, however, growing evidence that resistance and proprioceptive exercises are more effective than movement-based exercises alone^23-27^. In recent years there has been an emphasis on the need to restore normal scapular kinematics by improving strength, balance, and flexibility of muscles which control scapular position and motion^28-31^. The recommendations for duration of conservative treatment before surgery is contemplated has varied widely in the literature. In most studies, it has ranged from twelve months to eighteen months^17^.

Despite the fact that there has been so much research on exercise therapy for SAIS, there still remains insufficient evidence to support or disprove specific exercise programs for the treatment of patients with SAIS.

**B. Operative treatment**

Many surgeons resort to operative treatment when conservative treatment fails to provide pain relief. The commonly performed operation is an anterior acromioplasty with resection of the coracoacromial ligament.

Anterior acromioplasty can be performed with an open technique which was first described by Neer^2^ or by the arthroscopic technique described by Ellman^32^. Other surgeons carry out arthroscopic subacromial decompression where decompression of the subacromial space is done by removing bone spurs and soft tissue.

Several authors have reported good outcome in 73% to 93% of the patients who were treated with open acromioplasty^33-36^. Similarly, there are several authors who have reported good results with arthroscopic acromioplasty^37-43^.

The worst results for arthroscopic acromioplasty were reported by Hawkins *et al*^44^. They reviewed the results of 110 consecutive arthroscopic acromioplasties in patients who were followed-up for at least two years. Satisfactory results were only seen in 46% of the patients. The authors were of the opinion that open decompression yields superior results.

### Complications of Arthroscopic Acromioplasty

Complications are low at between 0.76% to 6.5% with arthroscopic acromioplasty^45, 46^. The most common complication has been insufficient removal of bone leading to a need for a revision operation. Acromion fracture as a complication has also been reported^47^.

Infection rates of between 0.04% to 3.4% have been reported for arthroscopy of the shoulder^48^. Musculocutaneous nerve, median, ulna, and radial nerve injuries can occur during shoulder arthroscopy^49^.

Several reports of devastating complications with beach chair position for shoulder arthroscopy have been reported. Pohl and Cullen^50^ reported four cases of arthroscopic shoulder surgery that resulted in one death and severe brain damage in three others. Ophthalmoplegia, stroke, brain death and loss of vision has also been reported^51, 52^.

Fractures of the clavicle, acromion, and humerus can also occur during shoulder arthroscopy^53^. Stiffness of the shoulder is probably the most common complication after shoulder surgery. It leads to significant morbidity, loss of function and disability. The incidence of shoulder stiffness after shoulder arthroscopy varies between 2.8%^46^ to 15%^54^.

Chondrolysis following shoulder arthroscopy is a rare but devastating complication. Intraarticular pain pumps using bupivacaine have been implicated in the causation of chondrolysis^55^. Thermal probes used during shoulder surgery are known to raise the intraarticular fluid temperatures to above 45ºC which can cause chondrocyte death^56^.

Another devastating complication of arthroscopic shoulder surgery is avascular necrosis of the humeral head^57-59^.

### Is Subacromial Impingement Syndrome a Medical Myth?

All these years the Neer concept of “impingement” has been accepted as the cause of rotator cuff disease and also formed the basis for clinical testing as well as describing radiographic and magnetic resonance imaging (MRI) changes.

Over the years, contact with the coracoid, superior glenoid, posterior and superior labrum were also implicated in the genesis of shoulder impingement syndrome^8^.

In recent years, however, impingement as a cause of rotator cuff disease has been questioned. Rotator cuff disease is now believed to be a form of tendinopathy similar to tendinopathies in other parts of the body. It is believed to be overuse tendinopathy and not a form of tendinitis. Histologically the findings are that of a failed healing response with little or no evidence of inflammation. Histological examination of the tendons show abnormalities of tenocytes proliferation, intracellular abnormalities in the tenocytes, collagen fibres disruption and an increase in the non-collagenous matrix^8^. Some of these abnormalities are characteristic of changes due to ageing. Physical loading, infection, smoking, vibration, injury, genetic factors, and use of fluoroquinolone antibiotics are factors which are known to produce such histologic features^13^.

The rotator cuff tendons have few nerve fibres. Mechanoreceptors and free nerve endings are found in the superior, middle, inferior, and posterior glenohumeral ligaments. They are also found in the coracoclavicular, and coracoacromial ligaments. Only the outer half of the glenoid labrum has nerve endings. Large numbers of free nerve endings are found in the subacromial bursae^60^.

The rotator cuff tendons play a role in generating pain through some indirect mechanism where some peptides or transmitters produced by the degenerated tendon initiate a pain response from the pain fibres in the bursa, ligaments of the joint and the joint capsule^61^.

There is no direct relationship between the presence of a rotator cuff tear and the presence of pain. Patients with large rotator cuff tears can have no pain while some patients with small tears can have severe pain^8^. Studies show that patients with failed rotator cuff repair obtain pain relief from surgery which means that rotator cuff tendon healing to bone is not necessary for a good surgical outcome. It also means that impingement is not the cause of the pain. Some other mechanism is responsible for the pain^8^.

Since the impingement theory has become outdated, the surgical treatment should have no role in the treatment of shoulder pain. Some authors have even proposed that the impingement syndrome should be renamed as anterolateral shoulder pain syndrome^8^. This new concept which refutes the existence of impingement is supported by several randomised controlled trials^62-66^ which show no supremacy of surgery over conservative treatment.

The most outstanding study was the one by Beard *et al*^62^. They carried out a multicentre, randomised, placebo-controlled, three-group trial at 32 hospitals in the UK. Included in the trial were patients with subacromial pain of three months duration who had completed conservative treatment and had at least one steroid injection and were eligible for arthroscopic surgery. Patients with full-thickness rotator cuff tear were excluded. They randomly assigned 106 patients to decompression surgery group, 103 patients to arthroscopy only group and 104 patients to no treatment group. At six months, Oxford Shoulder Score data was available for 90 patients in the decompression group, 94 patients in the arthroscopy group, and 90 patients in the no-treatment group. There was no difference in the mean Oxford Shoulder Score between the decompression and arthroscopy group at six months. Both surgical groups showed a small benefit over no treatment but these differences were not clinically significant. Surgical decompression offered no extra benefit over arthroscopy only. The authors believe that the difference between the surgical groups and no treatment group may be the result of a placebo effect or post-operative physiotherapy which the surgical group had. The authors questioned the value of subacromial decompression for shoulder pain.

Karjalainen *et al*^67^ carried out Cochrane systematic review of the literature to assess the benefits and harms of subacromial decompression surgery as compared with placebo, no intervention or non-surgical interventions in patients with subacromial impingement and rotator cuff disease. Patients with full-thickness rotator cuff tears were excluded.

The authors concluded that the evidence available does not support the use of subacromial decompression in the treatment of rotator cuff disease in patients with painful shoulder impingement. High-quality evidence shows that subacromial decompression does not provide clinically important benefit as compared to placebo as far as pain, function and health-related quality of life is concerned.

Physiotherapy provides good outcome which is comparable to that achieved with surgery. Conservative treatment also avoids the costs and the complications which are associated with surgery. Some of the complications associated with shoulder arthroscopy are devastating though rare.

## Conclusion

Subacromial impingement syndrome (SAIS) of the shoulder has been known for a long time and is probably the most common disorder of the shoulder. Several hypotheses have been put forward to describe the pathogenesis of SAIS but no clear explanation has been found. Two mechanisms have been described for impingement syndrome, the extrinsic and the intrinsic mechanism. The intrinsic mechanism theories are gaining popularity in recent years. The various shoulder tests used to diagnose SAIS have poor specificity with an average of about 50%. Meta-analysis shows that neither the Neer sign nor the Hawkins sign has diagnostic utility for impingement syndrome. Several randomised controlled trials have shown that the outcome of treatment of SAIS by surgery is no better than conservative treatment. Physiotherapy alone can provide good outcome which is comparable to that achieved with surgery without the costs and complications associated with surgery.

Since decompression with surgery does not provide additional benefits compared to conservative treatment for patients with SAIS, the impingement theory has become antiquated and surgical treatment should have no role in the treatment of such patients. There are calls by some to abandon the term impingement syndrome and rename it as anterolateral shoulder pain syndrome. There are others who have called SAIS as a clinical illusion^14^.
